# Bilateral thalamic stroke due to occlusion of the artery of Percheron in a patient with patent foramen ovale: a case report

**DOI:** 10.4076/1752-1947-3-7392

**Published:** 2009-09-15

**Authors:** Raúl López-Serna, Patricia González-Carmona, Manuel López-Martínez

**Affiliations:** 1 Division of Neurosurgery, Instituto Nacional de Neurología y Neurocirugía, México, DF, Mexico; 2 Emergency Department, Instituto Nacional de Neurología y Neurocirugía, México, DF, Mexico

## Abstract

**Introduction:**

Bilateral thalamic infarcts are rare presentations of stroke. They are the result of a complex combination of risk factors and a predisposing vessel distribution. The artery of Percheron, characterized by a single arterial trunk that irrigates both paramedian thalamic regions, can be occluded as a result of embolic diseases leading to bilateral paramedian thalamic infarcts. Clinical and image findings of this uncommon form of posterior circulation infarct are presented along with their anatomic and pathophysiologic correlates.

**Case presentation:**

A 27-year-old Mexican man with no relevant medical history was admitted to hospital after he was found deeply stuporous. On admission, an urgent neuroimaging protocol for stroke, including magnetic resonance imaging and magnetic resonance imaging angiography, was performed. The scans revealed symmetric bilateral hyperintense paramedian thalamic lesions consistent with acute ischemic events. The posterior circulation was patent including the tip of the basilar artery and both posterior cerebral arteries, making the case compatible with occlusion of the artery of Percheron. Further evaluation with an aim to define the etiology revealed a patent foramen ovale as the cause of embolism.

**Conclusion:**

Bilateral thalamic infarcts are unusual presentations of posterior circulation stroke; once they are diagnosed by an adequate neuroimaging protocol, a further evaluation to define the cause is necessary. Cardioembolism should always be considered in relatively young patients. A complete evaluation should be conducted by an interdisciplinary team including neurologists, cardiologists and neurosurgeons.

## Introduction

Although infarcts restricted to the thalamus were reported for the first time more than 100 years ago by Dejerine and Roussy [1], they remain a rare presentation of stroke and account for only 11% of all vertebrobasilar infarcts [2]. Bilateral involvement has been reported in a limited number of cases and results from a combination of predisposing factors and anatomic variations [3]-[9].

**Figure 1:**
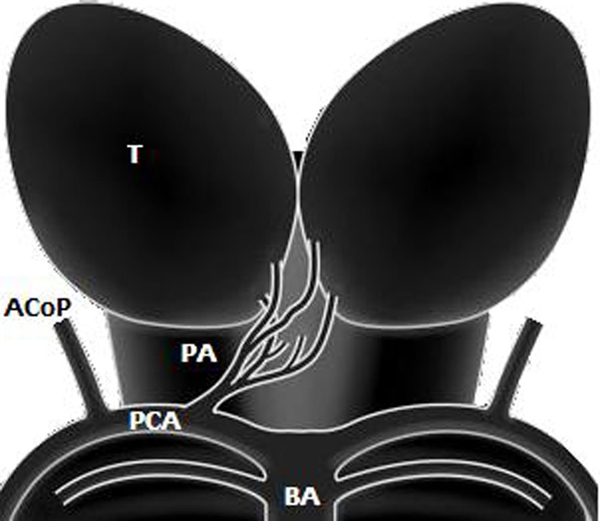
**Schematic representation of type B artery of Percheron emerging from the right first segment of the posterior cerebral arteries**. PA = type B artery of Percheron, T = thalamus, AcoP = posterior communicating artery, PCA = posterior cerebral artery, BA = basilar artery.

The thalamic arterial supply arises from perforating vessels with a complex distribution [10]. The paramedian thalamic territory is the median part of the thalamus including the intralaminar nuclei and most of the dorsomedian nucleus. It is supplied by the paramedian arteries, usually emerging directly from the first segment of posterior cerebral arteries (P1 segment) on both sides; however, in one-third of human brains, these originate from a single pedicle (Figure 1) known as the type B artery of Percheron [11,12]. Midbrain infarcts may result after occlusion of the artery of Percheron and they are usually limited to periaqueductal gray matter and affect the oculomotor and reticular nuclei.

Strokes limited to paramedian territories account for about 22% to 35% of all thalamic infarcts [13,14], and their most frequent etiology is cardioembolism [15]. The incidence of bilateral infarcts limited to this vascular territory has not been established.

## Case presentation

A 27-year-old Mexican man was admitted to our hospital after he was found in a deep stupor. He had no previous history of disease and did not smoke or take alcohol or illicit drugs. On admission, his vital signs were normal (blood pressure 125/70 mmHg, breath rate 18/minute, heart rate 78/minute, axillary temperature 37.2°C). On neurologic examination, we found ptosis, arreflectic mydriasis and exophthalmos of the right eye, suggesting that the right oculomotor nerve was affected. The left eye opened to the Foix maneuver with normal pupil reflexes. We further found symmetric facial responses and withdrawal of both arms and legs to painful stimuli. All brainstem reflexes were patent and Hoffman-Trömner and Babinski reflexes were negative on both sides. All blood tests were normal and illicit drug and toxic profiles were negative. The laboratory test results were as follows: complete blood cell count: hemoglobin 15.3, platelets 371; cholesterol profile: cholesterol 177 mg/dl, triglycerides 296, high-density lipoprotein (HDL) 35 mg/dl, low-density lipoprotein 83 mg/dl, ratio of cholesterol to HDL 5.1; anti-beta-2 glycoprotein I antibodies, anticardiolipin (immunoglobin G and M), activated partial thromboplastin time, Venereal Disease Research Laboratory, Factor V Leiden, activated protein C and S tests were all negative.

**Figure 2:**
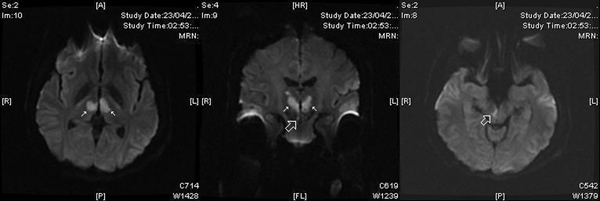
**Axial (right and left) and coronal (center) trace diffusion-weighted magnetic resonance images, obtained 20 hours after the onset of symptoms, show bilateral thalamic areas of high signal intensity (white arrows) compatible with acute bilateral paramedian thalamic infarcts**. Rostral midbrain infarct is limited to right periaqueductal gray matter (empty white arrows).

An urgent neuroimaging protocol for stroke including magnetic resonance imaging (MRI) and magnetic resonance image angiography (angio-MRI) was carried out. Axial and coronal trace diffusion-weighted images obtained 20 hours after the onset of symptoms showed bilateral areas of high signal intensity compatible with thalamic infarcts restricted to both paramedian thalamic territories and right periaqueductal gray matter (Figure 2). The posterior circulation was patent on the angio-MRI, including the tip of the basilar artery and both posterior cerebral arteries. Further evaluation with an aim of defining the etiology of the stroke revealed a patent foramen ovale on transesophageal echocardiography with spontaneous passage of contrast bubbles from the right auricle to the left cavities. Clot formation was found in the wall of the right auricle.

The state of consciousness spontaneously resolved during the third week after the ictus, although fluctuant periods of somnolence and obtundation continued. Administration of modafinil (100 mg twice a day) markedly improved his state of alertness, evidencing important neuropsychologic impairment with hyperphagia, anosognosia and emotional lability with depressive symptoms. There was improvement in the right third cranial nerve function with adequate eye opening and orthophoric condition, although bilateral limitation in vertical gaze movement was persistent. The patient was discharged after two weeks of hospitalization and was being followed up by our outpatient clinic at the time of writing. He began medical treatment with oral anticoagulation and was referred to a national cardiology center for treatment of his congenital cardiopathy. No other embolic events have occurred while there is a pending procedure of percutaneous closure using an Amplatzer device.

## Discussion

Strokes affecting both paramedian thalamic territories are unusual and may lead to a suspicion of an occlusion of a single arterial trunk known as the artery of Percheron. Although not visible on angio-MRI, the presence of this anatomic variant must be suspected when bilateral symmetric paramedian thalamic infarcts are revealed on image studies in the context of a patent basilar artery and posterior cerebral arteries.

The clinical pattern of this unique presentation of posterior circulation stroke usually consists of varying levels of decreased consciousness and neuropsychologic impairment. In most cases, the cognitive and behavioral changes become obvious when consciousness resumes [16].

**Figure 3:**
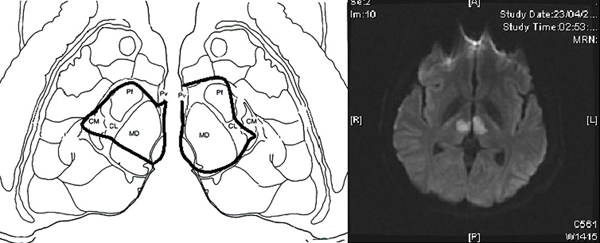
**Schematic representation of the affected thalamic nuclei in this case compared with an axial diffusion-weighted magnetic resonance image of the same patient**. The black line limits the area of infarction in both paramedian regions. CM = centromedian, Pf = parafascicularis, Pv = paraventricular, CL = central lateral, MD = dorsomedialis.

An understanding of thalamic anatomy is important to explain the pathophysiology of bilateral paramedian thalamic infarction. Paramedian nuclei consist mainly of a dorsomedian nucleus and intralaminar nuclei. The intralaminar nuclei consist of parafascicular, centromedian, central medial, paracentral and central lateral nuclei. Smaller nuclei of the 'midline', such as the paraventricular, rhomboid and reunions nuclei, are also included in the intralaminar group (Figure 3). Both nuclear groups are characterized by important and reciprocally activating connections with the anterior, orbitofrontal and medial prefrontal cortices through the thalamic peduncles [17,18], thus explaining the neuropsychiatric impairment and the loss of self-activation characteristic of paramedian infarctions. The rostral midbrain can also be involved after occlusion of the artery of Percheron. The initial presence of right mydriasis, ptosis and exophthalmos are all suggestive of an effect at this level due to the periaqueductal gray matter being affected, where the third cranial nerve nuclei are located. The recovery of function in our patient strongly suggests the presence of collateral midbrain circulation from mesencephalic branches emerging from the inferior and middle rami of the P1 segment. Patent vertical gaze limitation has been reported as part of thalamic syndromes, perhaps related to a remnant affecting the rostral interstitial nucleus of the medial longitudinal tract, precisely located between the diencephalon and the midbrain.

## Conclusion

In relatively young patients with no vascular risk factors, congenital cardiopathies must be taken into account as important possible causes of embolism. Among the main causes of embolic disease, the patent foramen ovale has previously been reported as a frequent etiology of stroke in young people [19]. Several short-numbered series and isolated case reports have been published about bilateral paramedian thalamic infarcts. To the best of our knowledge, our report is on the youngest case ever reported.

## Abbreviations

angio-MRI: magnetic resonance image angiography; HDL: high-density lipoprotein; LDL: low-density lipoprotein; MRI: magnetic resonance imaging.

## Consent

Written informed consent was obtained from the patient for publication of this case report and any accompanying images. A copy of the written consent is available for review by the Editor-in-Chief of this journal.

## Competing interests

The authors declare that they have no competing interests.

## Authors' contributions

LSR selected the case and drafted the manuscript. LSR and GCP cared for the patient and performed the investigation that led to the diagnosis. LMM analyzed and interpreted the patient data and neuroimaging protocol.
